# Silica-Lipid Hybrid Microparticles as Efficient Vehicles  for Enhanced Stability and Bioaccessibility of Curcumin

**DOI:** 10.17113/ftb.57.03.19.6035

**Published:** 2019-09

**Authors:** Yudi Ma, Qiang Wang, Dantong Wang, Juan Huang, Rui Sun, Xinyu Mao, Yuan Tian, Qiang Xia

**Affiliations:** 1 School of Biological Science and Medical Engineering, State Key Laboratory of Bioelectronics, Southeast University, No.2, Sipailou Street, 210096 Nanjing, PR China; 2 National Demonstration Center for Experimental Biomedical Engineering Education, Southeast University, No. 2, Sipailou Street, 210096 Nanjing, PR China; 3 Collaborative Innovation Center of Suzhou Nano Science and Technology, No. 150 Renai Road 215123 Suzhou, PR China

**Keywords:** curcumin, silica-lipid hybrid microparticles, antioxidant activity, bioaccessibility, storage stability

## Abstract

Curcumin is an active ingredient with multiple functions, but its application is often restricted due to its poor water solubility, weak stability, and consequently low bioaccessibility. Based on this, the aim of this work is to develop a new vehicle to overcome these restrictions. Here we developed a curcumin-loaded nanoemulsion and then curcumin-loaded silica-lipid hybrid microparticles through emulsification and vacuum drying, respectively. The loading of curcumin in the nanoemulsion and microparticles was (0.30±0.02) and (0.67±0.02) %, respectively. FTIR and XRD analyses of microparticles revealed that curcumin was encapsulated in porous, amorphous silica. *In vitro* antioxidant activities showed that the encapsulation would not affect the antioxidant activity of curcumin. *In vitro* simulated digestion indicated that nanoemulsion and microparticles had higher curcumin bioaccessibility than the control group. The storage stability of microparticles remained the same during 6 weeks in the dark at 4, 25 and 40 °C. Moreover, the microparticles had a better chemical stability than nanoemulsion under the light. The cell viability was over 80% when the concentration of nanocarriers was less than 45 μg/mL. Hence, the microparticles could be a promising means to load curcumin and improve its solubility, light stability and bioaccessibility.

## INTRODUCTION

Curcumin is extracted from the rhizome of *Curcuma longa*, which has extensive pharmacological and biological activities. In traditional medicine, curcumin has been valuable in the treatment of various inflammation and other diseases for centuries (*1*). In recent years, a number of researchers have focused on curcumin and it has been well-documented that curcumin possesses antioxidant, antimicrobial, anti-inflammatory and anticancer activities (*2*-*4*). Many reports have confirmed the potential of curcumin in the treatment of diabetes, rheumatoid arthritis, neurodegenerative diseases and cardiovascular diseases (*5*-*7*). Furthermore, curcumin is approved as a Generally Recognized as Safe compound by the U.S. Food and Drug Administration (*8*), indicating the potential value in food and health care products because of its high security and biological activities.

Despite a variety of advantages, practical applications of curcumin are difficult because of its low water solubility, chemical instability and rapid metabolism in the gastrointestinal tract (*8*, *9*). These shortcomings ultimately lead to inadequate absorption and low bioavailability of curcumin. To solve these problems, many strategies have been developed which include chemical/physicochemical and physicomechanical methods (*10*). Among them, the lipid-based nanoparticle delivery systems are widely recognized due to their advantages and application prospect. The most common types of nanoparticles include nanoemulsion, solid lipid nanoparticles, nanostructure lipid carriers, lipo-somes, self-emulsifying drug delivery systems and so on. These nanoparticles can improve the water solubility and bioaccessibility of curcumin (*11*). Besides, the nanocarriers can encapsulate the curcumin molecules inside the cavities to protect curcumin from degradation and improve stability (*12*). Several studies show that the nanocurcumin has higher antioxidant and antimicrobial activities than raw curcumin (*13*, *14*). Furthermore, other activities of curcumin such as anticancer activity should not be affected. Researchers have found that liposomal curcumin could suppress the growth of pancreatic carcinoma and demonstrated antiangiogenic effects *in vivo* (*15*). Another research reveals that curcumin in solid lipid nanoparticles has xenograft-targeting effect and enhances the tumour inhibition efficiency significantly from 19.5 to 69.3% (*16*). These results indicate that the activity of nanocurcumin is tantamount to or better than raw curcumin.

Although these lipid-based nanoparticles have many advantages, they still face the challenges of physical stability and long-term storage stability. Solidification of liquid formulation, such as dry emulsion, is considered a promising approach to solve these problems, which can improve the physicochemical stability during long-term storage. Spray drying (*17*), lyophilisation (*18*), vacuum drying (*19*) and physical adsorption (*20*) are the common solidification methods to eliminate the water phase from the liquid emulsion. Silica-lipid hybrid (SLH) microparticles are a kind of solid system based on silica particles and oil-in-water emulsion, which were reported for the first time by Simovic *et al.* (*21*) and Tan *et al.* (*22*). The researches show that the SLH microparticles significantly improve the solubility and stability of the water-insoluble drugs, and also enhance the oral absorption of drugs in experimental animals by simulating food effects (*21*-*23*). In another study (*24*), researchers found that the ibuprofen in SLH microparticles prepared by spray drying has higher solubility than the control group during a two-step dissolution and its bioavailability increased nearly 1.95-fold compared to the commercial tablets in clinical trials. Furthermore, the safety assessments reveal negligible acute side effects associated with the blank and ibuprofen in SLH formulation (*24*). These results indicate that microparticles are effective and are safe for oral consumption, which is a promising method to improve the oral absorption of water-insoluble substance.

Various methods have been developed to improve the water solubility and bioavailability of curcumin in recent years, but there is little research of the effect of curcumin-loaded silica-lipid hybrid microparticles. In the current study, curcumin nanoemulsion was developed and the optimum formulation was selected through orthogonal experiment. Then the microparticles were prepared with nanoemulsion in a two-step process: production of silica-stabilised nanoemulsion and vacuum drying. The physicochemical properties, antioxidant ability, bioaccessibility, lipid digestion property, and storage stability of the two samples were examined to investigate the similarities and differences between the microparticles and nanoemulsion. This investigation will provide a new perspective to solve the problems of curcumin and promote its application in more fields.

## MATERIALS AND METHODS

### Materials

Curcumin (95%) was purchased from Sciphar Natural Products Co. Ltd. (Shaanxi, PR China). Octyl and decyl glycerate (ODO) was obtained from Henan Zhengtong Food Technology Co. Ltd. (Henan, PR China). Tween 80 and Tween 60 were provided by Guangzhou Runhua Chemical Co. Ltd. (Guangzhou, PR China). Phosphatidylcholine 60 (PC 60) was produced by Aikang Fine Chemical Co. Ltd. (Shanghai, PR China). Hydrophilic fumed silica (Aerosil 380) was provided by Evonik Degussa (Essen, Germany). Povidone K30 (PVP (polyvinylpyrrolidone) K30) was purchased from Star-Tech & JRS Specialty Products Co. Ltd. (Chongqing, PR China). Carboxymethylcellulose sodium (CMC-Na) was obtained from Yuhe Food Additive Co. Ltd. (Zhengzhou, China). All the ingredients were of food grade.

The 2,2-diphenyl-1-picrylhydrazyl (DPPH) free radical was provided by Tokyo Chemical Industry Co. Ltd. (Tokyo, Japan), and 1,1,3,3-tetraethoxypropane (97%) was purchased from Shanghai Macklin Biochemical Co. Ltd. (Shanghai, PR China). Phosphotungstic acid, potassium bromide (KBr), trichloroacetic acid (TCA) and 2-thiobarbituric acid (TBA) (≥98.5%) were from Sinopharm Chemical Reagent Co. Ltd. (Shanghai, PR China). Pepsin, pancreatin and bile extract were from Sigma-Aldrich, Merck (St. Louis, MO, USA). DMEM, fetal bovine serum (FBS) and penicillin-streptomycin were purchased from Cellmax Cell Technology Co. Ltd. (Beijing, PR China). All other chemicals used were of analytical grade without further purification.

### Preparation of curcumin nanoemulsion

Several mass fractions of Tween 80 (2.0–5.0%), Tween 60 (2.0–5.0%) and PC 60 (2.0–5.0%) were dissolved in 10% ODO at 70 °C, followed by the addition of 0.3% curcumin raw material. PVP K30 0.6–1.2% was added to the oil phase after curcumin was completely dissolved. Then, Milli-Q water (Arium pro Basic, Sartorius, Goettingen, Germany) (73.5–83.1%) was added to the oil mixture as the continuous phase. The mixture was stirred for 10 min to form oil in water (o/w) emulsion.

### Optimisation of curcumin nanoemulsion

The composition of curcumin nanoemulsion was optimized by orthogonal experiment design with 5 factors (A-E) at 4 levels respectively: A=temperature (25, 40, 55 and 70 °C) of the preparation, and the mass fractions of B=Tween 80 (2, 3, 4 and 5%), C=Tween 60 (2, 3, 4 and 5%), D=PC 60 (2, 3, 4 and 5%) and E=PVP K30 (0.6, 0.8, 1.0 and 1.2%). Emulsion stability index (ESI) was chosen as evaluation index (*25*). The emulsion was stored at room temperature for 48 h after preparation, and then the data were measured to calculate the ESI as follows:
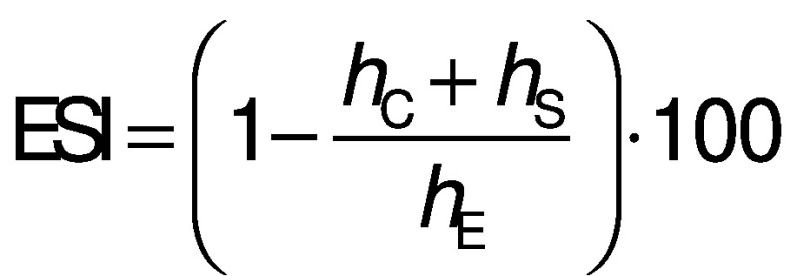
where *h*_C_ is the height of creamed phase, *h*_E_ is the total height of the emulsion and *h*_S_ is the height of sedimentation phase.

### Preparation of curcumin silica-lipid hybrid microparticles

According to the method of Tan *et al.* (*22*), the microparticles were prepared using a two-step process: the production of silica-stabilised nanoemulsion and vacuum drying. Curcumin nanoemulsion was prepared as described in the section *Preparation of curcumin nanoemulsion*. The silica was added into o/w nanoemulsion in different silica-to-lipid ratio by stirring constantly. The silica-stabilised emulsion was then vacuum-dried (DZF-6090; Jinghong, Shanghai, PR China) under a pressure of 100 Pa at 50 °C to form solid powder. Drying lasted 4 h. Different silica-to-lipid mass ratios (1:1, 1:2 and 1:3) were chosen to explore the effect of different additions on the formulation.

### Morphological analysis

#### Transmission electron microscopy

The morphological characteristics of curcumin nanoemulsion were studied by transmission electron microscope (TEM model JEM-2100; JEOL, Tokyo, Japan). The diluted nanoemulsion sample was dropped onto carbon-coated grids and then negatively stained with 2% (*m/V*) phosphotungstic acid. Subsequently, the excess liquid was wiped dry by filter paper and the sample was dried at room temperature.

#### Scanning electron microscopy

The shape and surface characteristics of hydrophilic fumed silica having a specific surface area of 380 m^2^/g (Aerosil 380) and curcumin silica-lipid hybrid microparticles were explored by scanning electron microscope (SEM model Ultra Plus; Zeiss, Jena, Germany). The powder sample was fixed on a slide using double-sided adhesive tape and the characterized by a secondary electron detector.

#### Measurements of particle size and polydispersity index

The average redispersed particle sizes and polydispersity index (PDI) of the formulations were investigated by dynamic light scattering (DLS) technique (Zetasizer Nano ZS 90; Malvern Panalytical Ltd, Malvern, UK) at a fixed scattering angle of 90° and room temperature. Before measurement, 0.05 g curcumin nanoemulsion or silica-lipid hybrid microparticles were dispersed in 10 g Milli-Q water (Arium pro Basic, Sartorius). The aqueous dispersion was centrifuged (TGL-16; Xiangyi, Changsha, PR China) at 633×*g* for 10 min in order to precipitate the silica microparticles. Each sample was measured three times.

#### Loading of curcumin

The loading of curcumin in nanoemulsion and microparticles was measured by UV/Vis spectrophotometer (755B; Jinghua, Shanghai, PR China) at the wavelength of 425 nm. A mass of 0.1 g nanoemulsion or microparticles was mixed with 9.9 g ethanol to extract the curcumin from the formulations. The ethanol extractions of curcumin were diluted 100 times and centrifuged (TGL-16; Xiangyi) at 5595×*g* for 10 min before measurement. Curcumin loading was calculated by the following formula:


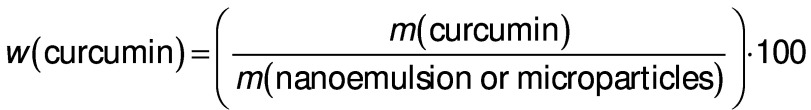


#### X-ray diffraction

The samples were characterized by X-ray diffraction instrument (D8 Discover; Bruker GmbH, Mannheim, Germany) with CuK_α_ radiation at 30 mA and 40 kV. The diffraction pattern of Aerosil 380, curcumin silica-lipid hybrid microparticles, and the physical mixture of curcumin and Aerosil 380 was measured over a 2*θ* range of 5–60°. The scanning speed was 0.15 s/step with a step size of 0.02°.

#### Fourier transform infrared spectroscopy

Infrared spectra of curcumin, blank silica-lipid hybrid microparticles, curcumin silica-lipid hybrid microparticles, and the physical mixture of curcumin and blank microparticles (curcumin content was the same as microparticles) were investigated by FTIR spectrophotometer (Nicolet 6700; Thermo Scientific, Waltham, MA, USA). The samples were mixed separately with potassium bromide and ground into fine powder. The mixture was processed into a tablet by electric tablet press (DY-30; Keqi, Shanghai, PR China) under a force of 25 MPa for 10 s, and then analyzed in the range of 4000–400 cm^–1^.

#### In vitro antioxidant activity

##### DPPH scavenging activity

A volume of 100 μL of nanoemulsion and silica-lipid hybrid microparticles in ethanol (100 μL of ethanol as blank control group) with various mass fractions of curcumin were added to 3.9 mL DPPH ethanol solution, and then mixed evenly. The reaction was performed at ambient temperature in the dark. After 30 min of reaction, the absorbance of the mixture was measured at 517 nm by UV/Vis spectrophotometer (755B; Jinghua, Shanghai, PR China). The antioxidant activity was calculated as follows:


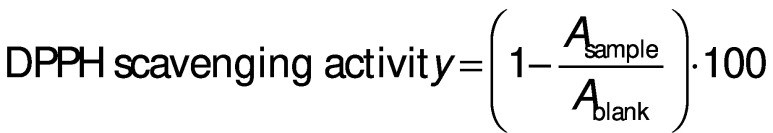


##### Anti-lipid peroxidation effects

Different concentrations of curcumin nanoemulsion and silica-lipid hybrid microparticles in Milli-Q water (Arium pro Basic, Sartorius) were prepared. A volume of 0.5 mL of the sample was mixed with 1 mL of 1% (*m/V*) soybean phospholipid solution (0.5 mL of Milli-Q water as blank), 1 mL of 0.2 M (pH=7.4) phosphate buffer solution (PBS) and 1 mL of 5 mM FeCl_2_ in the tubes. The mixtures were incubated at 37 °C for 60 min in the dark. Afterwards, 3 mL of TBA-TCA-HCl (containing 0.025 M thiobarbituric acid (TBA), 0.92 M TCA and 0.58 M HCl) were added to the mixtures to stop the reaction. The tubes were placed at 95 °C for 15 min then cooled in an ice bath. The supernatant was collected after centrifugation (TGL-16; Xiangyi) at 685×*g* for 10 min and the absorbance was measured at 532 nm. Thiobarbituric acid reactive substances (TBARS) inhibition rate was calculated as follows:


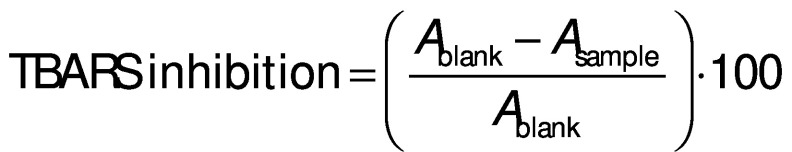


##### In vitro simulation of digestion

A two-step experiment was performed to simulate the gastric and small intestinal digestion of curcumin nanoemulsion and silica-lipid hybrid microparticles. Unformulated curcumin was suspended in 0.5% (*m/V*) CMC-Na solution as a control group (*26*). The experiment was done according to Huang *et al.* (*27*) with some modifications.

To test simulated gastric digestion, simulated gastric fluid (SGF) was prepared by adding 7 mL HCl (36.5%) and 2 g NaCl to 1 L Milli-Q water (Arium pro Basic, Sartorius), and then adjusting the pH to 1.2 with 1.0 M HCl. A volume of 10 mL of nanoemulsion diluent, aqueous dispersion of microparticles and curcumin CMC-Na suspension containing 1 mg/mL of curcumin was mixed with 10 mL of SGF, respectively. The mixture was incubated at 37 °C for 2 h with agitation at 100 rpm (EMS-30; Qianyue, Shanghai, PR China) after 32 mg of pepsin were added to it.

To test simulated intestinal digestion, 0.05 M potassium dihydrogen phosphate solution served as simulated intestinal fluid (SIF). Curcumin encapsulated in silica nanoparticles stabilised Pickering emulsion during storage and simulated digestion. The pH of the gastric phase was adjusted to 7.0 after simulated gastric digestion. Later, 10 mL of SIF containing 47.6 mg of pancreatin and 51.6 mg of bile extract were added to the mixture and incubated at 37 °C. At this point, the pH-stat titration served to maintain the pH of digestive juice at 7.0 by adding 0.1 M NaOH dropwise. The volume of NaOH was recorded at different time points during 2 h. The released free fatty acids (FFA) were calculated according to the following equation (*11*):


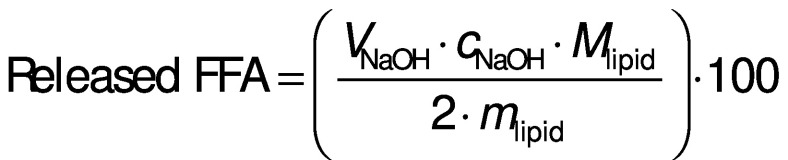


where *V*_NaOH_ is the total volume of NaOH (mL) used to neutralize the released FFA, *c*_NaOH_=0.1 M is the concentration of NaOH in the burette, *M*_lipid_ is the molecular mass of the oil in the formula (g/mol), *m*_lipid_ is the total mass (g) of lipid in the digestive juice. The particle size of the supernatant of the mixture was measured after each phase.

The *in vitro* bioaccessibility of curcumin was measured after simulated digestion. The digestive solution was centrifuged (TGL-16; Xiangyi) at 5595×*g* for 10 min and separated into two parts: one was the supernatant containing the mixed micelles, the other was the opaque sediment phase. The amount of curcumin in the supernatant was determined and the bioaccessibility was calculated as follows:


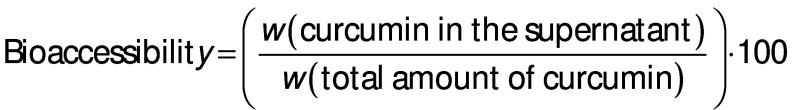


#### Storage stability

Curcumin nanoemulsion and silica-lipid hybrid microparticles were stored at 4, 25 and 40 °C (in the dark) and 25 °C under light for 6 weeks. Curcumin content was measured every week of the storage (ethanol solution of curcumin was used as a control). Also, the dispersion of particle size and appearance of microparticles were inspected.

#### Cell toxicity evaluation

L929 murine fibroblast cell line, purchased from the Type Culture Collection of the Chinese Academy of Sciences (Shanghai, PR China) was chosen for cell studies. Cells were cultured in DMEM medium supplemented with 10% FBS and 1% penicillin-streptomycin at 37 °C in a 5% CO_2_ incubator.

For the assessment of cell viability, the cells were plated in 96-well plates (100 μL/well) at a density of 10^4^ cells/mL and cultured for 12 h (blank group was only given an equal volume of cell culture medium without cells). The curcumin nanoemulsion, blank nanoemulsion, silica-lipid hybrid microparticles and blank particles were diluted to various concentrations (4, 8, 12, 20, 30, 45, 60, 80, 100 and 150 μg/mL) with culture medium and then added to 96-well plates (Goyoobiotech, Nanjing, PR China) (100 μL/well). Control and blank groups received 100 μL cell culture medium. The medium was removed and the cells were washed with PBS after 24 h of treatment. Then, 200 μL cell counting kit-8 (CCK-8; Goyoobiotech) solution (10% in culture medium) were added to the wells for further incubation for 1.5 h, and then the absorbance (*A*) was recorded by a microplate reader (K3; Thermo Scientific) at 450 nm. The cell viability was calculated as follows:


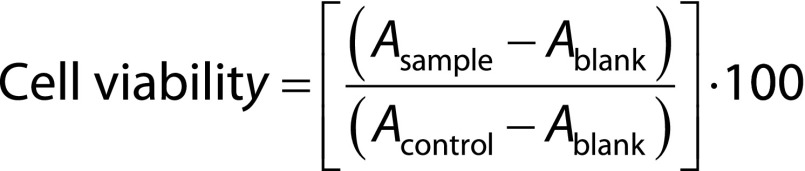


### Statistical analysis

All the experiments were done in triplicate. Results were expressed as mean value and standard deviation (mean±S.D.). The results were analyzed by ANOVA and *t*-test. Data were considered significant at p<0.05.

## RESULTS AND DISCUSSION

### Results of orthogonal experiment

Orthogonal experiment is a method to analyze the correlations of variables at different levels by an orthogonal table and statistical analysis, which can be used for the optimization of multiple formulae (*28*, *29*). The stability of o/w emulsion is closely related to many factors such as the preparation conditions and formulation composition. An orthogonal experiment was designed to optimize the formulation of curcumin nanoemulsion by testing the effects of preparation temperature and emulsifier mass fraction on the ESI. The results and analysis of orthogonal experiment are shown in Table 1. The sequence of effects on ESI to evaluate the preparation was: A (temperature)>D (PC 60)>E (PVP K30)>C (Tween 60)>B (Tween 80), which indicated that the temperature had significant effect on the stability of the formula and Tween 80 had little influence on the ESI of the o/w emulsion. Comprehensively considering the performance of each level, the optimum preparation conditions were A_4_B_4_C_3_D_2_E_1_. Based on the results, the optimal conditions for the preparation of nanoemulsion were ODO 10%, curcumin 0.3%, Tween 80 5%, Tween 60 4%, PC 60 3%, PVP K30 0.6% and Milli-Q water 77.1%, and preparation temperature 70 °C.

**Table 1:** The results of orthogonal experiment

No.	Factor					Cur/%	ODO/%	Water/%	Score
A/°C	B/%	C/%	D/%	E/%
1	25	2.0	2.0	2.0	0.6	0.3	10	83.1	33.93
2	25	3.0	3.0	3.0	0.8	0.3	10	79.9	31.30
3	25	4.0	4.0	4.0	1.0	0.3	10	76.7	30.97
4	25	5.0	5.0	5.0	1.2	0.3	10	73.5	32.77
5	40	3.0	4.0	5.0	0.6	0.3	10	77.1	31.50
6	40	2.0	5.0	4.0	0.8	0.3	10	77.9	30.90
7	40	5.0	2.0	3.0	1.0	0.3	10	78.7	35.47
8	40	4.0	3.0	2.0	1.2	0.3	10	79.5	46.97
9	55	4.0	5.0	3.0	0.6	0.3	10	77.1	65.10
10	55	5.0	4.0	2.0	0.8	0.3	10	77.9	59.23
11	55	2.0	3.0	5.0	1.0	0.3	10	78.7	19.39
12	55	3.0	2.0	4.0	1.2	0.3	10	79.5	33.17
13	70	5.0	3.0	4.0	0.6	0.3	10	77.1	78.33
14	70	4.0	2.0	5.0	0.8	0.3	10	77.9	27.53
15	70	3.0	5.0	2.0	1.0	0.3	10	78.7	58.40
16	70	2.0	4.0	3.0	1.2	0.3	10	79.5	70.07
k1	32.24	38.57	32.52	49.63	52.22				
k2	36.21	38.59	44.00	50.48	37.24				
k3	44.22	42.64	47.94	49.26	36.06				
k4	58.58	51.45	46.79	27.80	45.74				
R	26.37	12.88	15.42	22.68	16.16				

A=temperature (° C) of the preparation, B, C, D, E=mass fractions (%) of Tween 80, Tween 60, PC 60 and PVP K30, respectively, and ODO=octyl and decyl glycerate

### Curcumin silica-lipid hybrid microparticle formation

Microparticles with different silica-to-lipid mass ratios (1:1, 1:2 and 1:3) were prepared by vacuum drying. The sample with silica-to-lipid mass ratio 1:3 appeared as a thick, oily mixture after vacuum drying and there was no powder product formed. The sample with 1:2 ratio resulted in yellow agglomerated mass with poor powder mobility. The microparticles with silica-to-lipid mass ratio 1:1 formed dry and free flowing powder which was up to the standard.

### Physicochemical characterization of nanoemulsion and microparticles

**Fig. 1:**
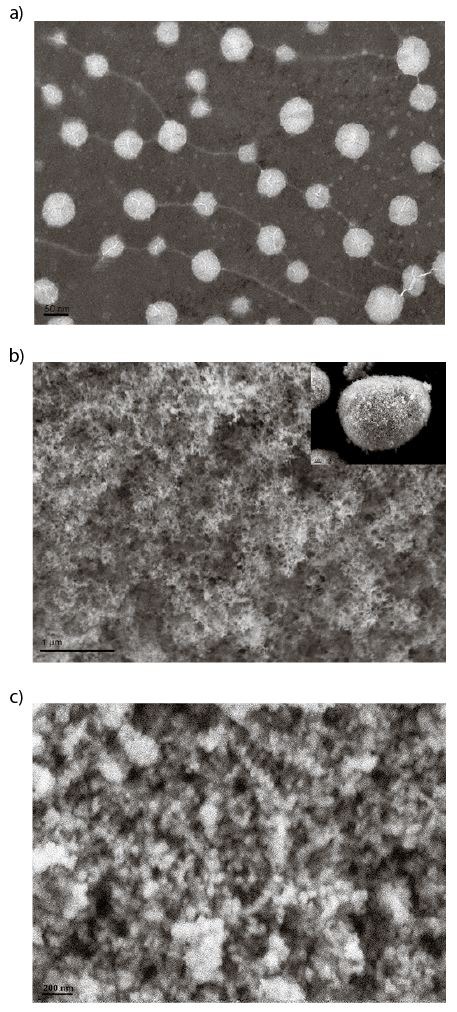
Transmission electron microscopic image of: a) curcumin nanoemulsion, and scanning electron microscopic images of: b) hydrophilic fumed sillica (Aerosil 380), and c) curcumin silica-lipid hybrid microparticles

Table 2 shows the particle size (redispersed particle size) and PDI of curcumin microparticles. The particle size of the nanoemulsion and microparticles was further analyzed by TEM. The hydrophobic tails of emulsifier molecules attach to hydrophobic core formed by the oil phase, while the hydrophilic heads protrude into aqueous phase, forming small spheroid particles dispersed in the aqueous phase. The spherical structure of nanoemulsion droplets is observable in Fig. 1a. The dynamic light scattering test showed that the particle size of nanoemulsion was (40.5±2.4) nm, which was substantiated by the diameter of particles in the TEM. PDI of nanoemulsion and microparticles was (0.16±0.02) and (0.26±0.02), respectively. The particle size and PDI of nanoemulsion were significantly smaller ((177.2±3.3) nm) than of microparticles. This could be ascribed to the oil droplet interfaces absorbed by tiny silica particles, leading to an increase in the particle size of the redispersed microparticles (*30*). The SEM images of Aerosil 380 and microparticles are shown in Fig. 1b and Fig. 1c, respectively. In a previous study (*31*), Aerosil 380 consisted of 7-nm primary particles, which indicates a porous internal structure with pore sizes ranging from 25 to 100 nm. The SEM image (Fig. 1b) showed a loose, porous and nearly spherical shape which gave the particles highly specific surface area and strong adsorption ability. Nevertheless, the microparticles (Fig. 1c) lost these features and had a rough surface, with microparticles formed when the oil phase was adsorbed onto the porous silica matrices. The results were consistent with the findings of Simovic *et al.* (*21*) where droplets were uniformly distributed and absorbed onto the surface of silica instead of forming a continuous film. In other study, Tan *et al.* (*22*) proposed that there could be redistribution and self-assembly of the Aerosil 380 particles from the continuous phase to the droplet surface and the inner wick during dehydration. The curcumin contents of nanoemulsion and microparticles were (0.30±0.02) and (0.67±0.02) %, respectively, indicating that curcumin loading increased after solidification.

**Table 2:** Appearance, size of dispersed particles and polydispersity index (PDI) of the microparticles at different temperatures and storage time

Temperature/ °C	*t*(storage)/ week	Appearance	*d*(dispersed particle)/nm	PDI
4	2	Yellow powder	178.0±1.8	0.26±0.02
	4	Yellow powder	178.0±2.0	0.26±0.02
	6	Yellow powder	178.8±1.3	0.25±0.03
25	2	Yellow powder	179.4±1.6	0.26±0.01
	4	Yellow powder	179.27±0.09	0.26±0.01
	6	Yellow powder	181.5±2.1	0.26±0.02
40	2	Yellow powder	180.7±2.3	0.26±0.01
	4	Yellow powder	179.0±3.0	0.263±0.009
	6	Yellow powder	179.3±2.0	0.27±0.01

### XRD spectra

**Fig. 2:**
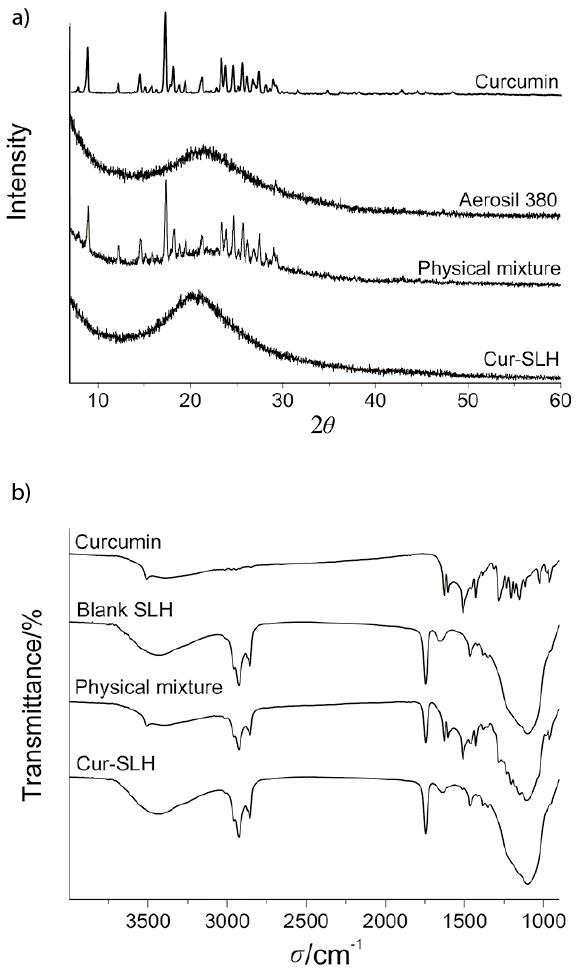
The spectra of: a) X-ray diffraction of curcumin, Aerosil 380, physical mixture of curcumin and Aerosil 380, and curcumin silica-lipid hybrid microparticles (Cur-SLH), and b) FTIR of curcumin, blank silica-lipid hybrid microparticles (Blank SLH), physical mixture of curcumin and blank SLH, and Cur-SLH microparticles

Crystalline or amorphous phase of curcumin in the silica-lipid hybrid microparticles was investigated by XRD analysis. The XRD spectra of curcumin, Aerosil 380, physical mixture of curcumin and Aerosil 380 and curcurmin microparticles are shown in Fig. 2a. Several sharp peaks in the diffractogram of the physical mixture of curcumin and Aerosil 380 indicated that curcumin existed in crystalline form. In contrast, the XRD spectrum of microparticles was similar to that of Aerosil 380 particles and there was no characteristic peak observed. The results suggested that curcumin was in an XRD-amorphous state in the microparticles.

### FTIR spectra

FTIR was chosen to investigate the interactions between curcumin and excipients in the solid state. The FTIR spectra of curcumin, blank silica-lipid hybrid microparticles, physical mixture of curcumin and curcumin silica-lipid hybrid microparticles are shown in Fig. 2b. A characteristic peak observed at 3508 cm^–1^ in the spectra of curcumin powder corresponded to the O–H stretching vibration, which was different from the broad peak of amorphous curcumin in the study of Li *et al*. (*32*). This phenomenon suggested a different molecular environment of hydroxyl groups between crystalline curcumin and amorphous curcumin. The peak at 1627 cm^–1^ was related to the C=O group and the band at 1509 cm^–1^ indicated the presence of C=C group. Meanwhile, these characteristic peaks still existed in the spectra of physical mixture, indicating no interaction between the curcumin and blank microparticles. However, the characteristic peaks disappeared in curcumin microparticles and the spectrum was similar to that of blank microparticles. Therefore, the results indicate that curcumin was incorporated in Aerosil 380 and remained in dissolved state in the microparticles (*33*, *34*).

### In vitro antioxidant activity of curcumin nanoemulsion and microparticles

#### DPPH free radical scavenging assay

**Fig. 3:**
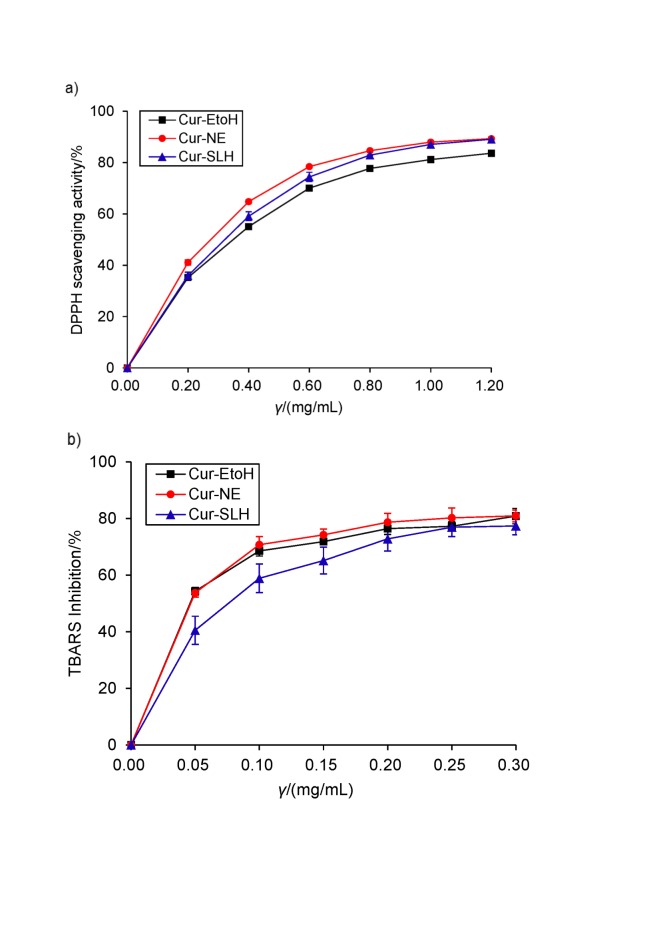
Results of: a) DPPH scavenging activity of curcumin ethanol solution (Cur-EtOH), curcumin nanoemulsion (Cur-NE) and curcumin silica-lipid hybrid microparticles (Cur-SLH). Results are shown as mean value±S.D. (*N*=3), and b) TBARS inhibition by Cur-EtOH, Cur-NE and Cur-SLH. Results are shown as mean value±S.D. (*N*=3)

DPPH is a stable radical which has been extensively applied in the analysis of antioxidant activity. Generally, DPPH signals decrease when the odd electron of DPPH radical becomes paired with hydrogen from a free radical scavenging antioxidant to form the reduced DPPH-H (*35*). In this study, the DPPH assay was used to explore the free radical scavenging activity of curcumin nanoemulsion and microparticles. According to the results in Fig. 3a, the nanoemulsion and microparticles showed a higher DPPH scavenging activity than curcumin ethanol solution. The DPPH scavenging activity of nanoemulsion was 87.98 and of microparticles 87.06%, which was higher than of control (81.17%) when the concentration was 1 mg/mL. It can be postulated that the nanoparticles protected curcumin from degradation and thus increased its antioxidant activity. In addition, the radical scavenging activity of microparticles was slightly lower than that of nanoemulsion when the concentration was lower than 1 mg/mL. Sari *et al*. (*36*) concluded that the encapsulation of silica not only reduces the degradation of curcumin but may also preserve its antioxidant activity. Hence, this result indicates that the free radical scavenging activity of curcumin was not affected by the encapsulation.

### Anti-lipid peroxidation

Lipid peroxidation is often responsible for biological damage, particularly the biological membrane of the brain or liver (*37*). Generally, malondialdehyde is one of the major products of lipid peroxidation, whose levels can be spectrophotometrically determined by forming a complex with the 2-thiobarbituric acid called TBA reactive substances (TBARS). The TBARS inhibition by curcumin nanoemulsion, silica-lipid hybrid microparticles and curcumin ethanol solution is shown in Fig. 3b. It was observed that the anti-lipid peroxidation capacities of nanoemulsion and microparticles increased in a dose-dependent manner, and the inhibition rate was over 50% at the concentration of 100 μg/mL. However, the TBARS inhibition by microparticles was slightly lower than of curcumin ethanol solution, which can be explained as the result of the slow release of curcumin from Aerosil 380.

### Results of in vitro simulation of digestion study

**Fig. 4:**
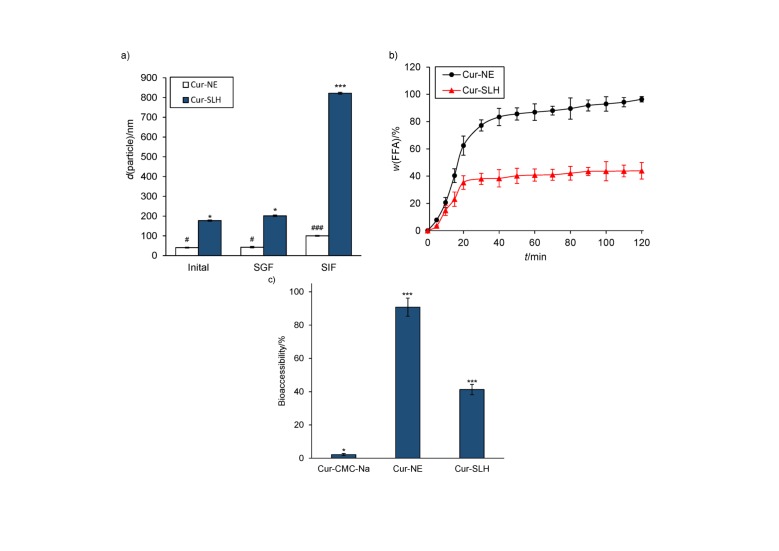
Results show: a) particle size of curcumin nanoemulsion (Cur--NE) and curcumin silica-lipid hybrid microparticles (Cur-SLH) before digestion in simulated gastric fluid (SGF) and simulated intestinal fluid (SIF). Results are shown as mean value±S.D. (*N*=3). Different symbols mean significant difference (p≤0.01), b) cumulative FFA released by Cur-NE and Cur-SLH during the simulated gastric digestion. Results are shown as mean value±S.D. (*N*=3), and c) bioaccessibility of curcumin from Cur-CMC-Na suspension (as a control group), Cur--NE and Cur-SLH after the simulated digestion. Results are shown as mean value±S.D. (*N*=3). ***Significantly different (p≤0.01)

A two-step experiment was used to investigate the simulated digestion of curcumin nanoemulsion and silica-lipid hybrid microparticles. The particle sizes of digestive juice were measured after each digestion phase by DLS technique. The changes in the particle size are observable in Fig. 4a. Nanoemulsion and microparticles before digestion had mean particle size of (40.5±2.4) and (177.2±3.3) nm, respectively. After gastric digestion, there was not much change in the particle size of the two samples. This result indicates that the structures of the two samples remained stable and the oil droplets were not destroyed during gastric digestion. However, after 2 h of intestinal digestion, the particle sizes of nanoemulsion and microparticles increased significantly to (100.0±2.3) and (822.0±4.7) nm, respectively. The augmentation of particle sizes suggested that droplets were destabilized in the process of simulated intestinal digestion. Previous research showed that bile salts can take the place of surfactant molecules from the oil-water interface, facilitate the binding of pancreatic enzymes to the oil-water interfacial layer and promote the lipid digestion, leading to the destabilization of emulsion (*38*, *39*). The complex reactions that occurred in the intestine resulted in the formation of mixed micelles with the ability to transport the encapsulated components to the surface of the enterocytes and enhance the absorption of nutrients (*40*).

During the intestinal digestion, oil droplets are broken down into free fatty acids and monoglycerides so the bioactive molecules can be released (*41*). In this study, the degree of lipid digestion from the nanoemulsion and microparticles was measured by instilling 0.1 M NaOH into digestive juice to neutralize free fatty acids. As shown in Fig. 4b, there was a rapid increase in the rate of free fatty acid release from nanoemulsion and microparticles in the first 30 min, followed by a more gradual increase from 30 to 120 min. However, there were some differences in the rate and extent of digestion between nanoemulsion and microparticles. The initial digestion rate of nanoemulsion was faster than that of microparticles, which can be attributed to the difference in the droplet size. A number of studies have found that the release rate of free fatty acids increases as the lipid droplet size decreases (*42*). Due to large specific surface area of small droplets, their contact with lipase molecule increased (*43*). Moreover, the final amount of free fatty acids released from the microparticles was lower than from nanoemulsion, which indicated that the microparticles can inhibit the release of oil droplets. The possible reason was that the oil-water interface was adsorbed by silica particles which led to the inhibition of oil release (*30*).

Bioaccessibility is defined as the fraction of the active ingredient which is solubilised from the food during simulated gastrointestinal digestion, so it is considered as an estimable method of oral bioavailability (*44*). The bioaccessibility of the samples was measured at the end of simulated intestinal digestion. As shown in Fig. 4c, there was a significant increase in bioaccessibility of nanoemulsion and microparticles compared to curcumin CMC-Na suspension, indicating that nanocarriers can improve the bioavailability of curcumin. However, it was obvious from the results that the bioaccessibility of nanoemulsion was higher than that of microparticles. Similarly, the bioaccessibility of the free fatty acids increased with the increase of their release. The difference in curcumin bioaccessibility between nanoemulsion and microparticles may be due to two reasons. Lower release of free fatty acids resulted in more undigested lipids, which entrap curcumin. Furthermore, there were not enough mixed micelles to dissolve curcumin (*45*).

### Storage stability of curcumin nanoemulsion and microparticles

**Fig. 5:**
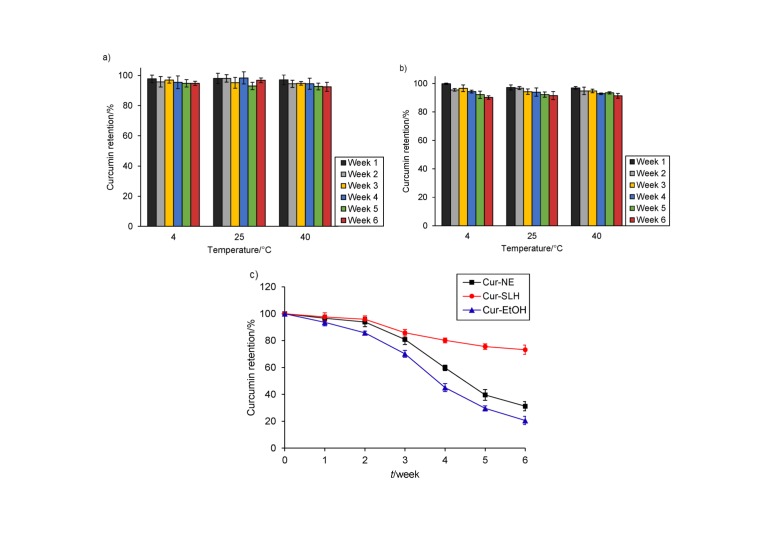
Retention ratios of curcumin in: a) curcumin nanoemulsion (Cur-NE), b) curcumin silica-lipid hybrid microparticles (Cur-SLH) during storage for 6 weeks at 4, 25 and 40 °C in the dark, and c) curcumin in ethanol solution (Cur-EtOH), Cur-NE and Cur-SLH during storage for 6 weeks at 25 °C under light. Results are shown as mean value±S.D. (*N*=3)

Stability is an important factor in examining the quality of the system. In this study, the retention ratios of curcumin in nanoemulsion, silica-lipid hybrid microparticles and ethanol solution was measured during 6 weeks of storage at different temperatures to investigate the chemical stability of the three samples. Fig. 5a shows that the retention ratios of curcumin in the nanoemulsion did not change significantly during storage at 4 and 25 °C, and changed slightly at 40 °C. On the whole, the nanoemulsion maintained good chemical stability during 6 weeks of storage. The same result was also obtained with microparticles (Fig. 5b). However, the samples stored in light behaved differently from those in the dark (Fig. 5c). During 6 weeks of storage, the curcumin retention ratios of nanoemulsion declined to 31.20%, which suggested the formulation was unstable when exposed to light and should be stored in the dark. The retention ratio of microparticles also decreased and remained at 73.23%. Even so, it was still clearly higher than that of nanoemulsion. This observation confirmed that solid preparations protected curcumin better when exposed to light. The possible reason was that the curcumin was encapsulated in the pores of Aerosil 380, which could reduce the influence of light on curcumin (*45*). Table 2 shows the dispersed particle size and appearance of curcumin microparticles during storage. The dispersed particle size and appearance have not changed significantly, which indicates a good physical stability.

### Cell toxicity evaluation of curcumin nanoemulsion and microparticles

**Fig. 6:**
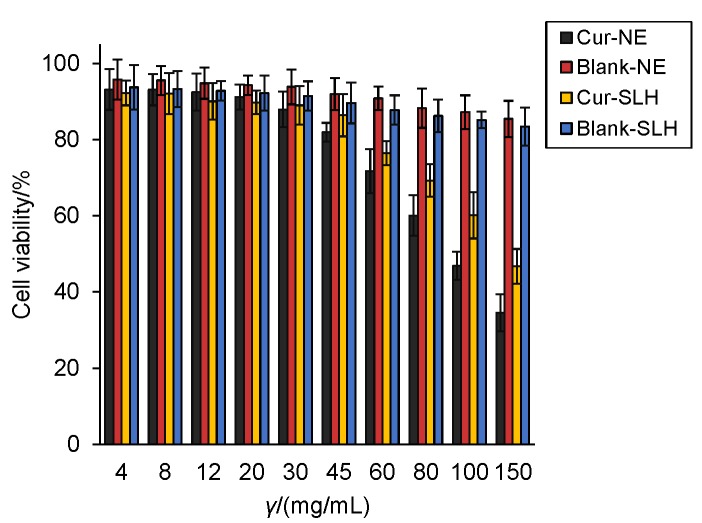
Cell viability of curcumin nanoemulsion (Cur-NE), blank nanoemulsion (Blank-NE), curcumin silica-lipid hybrid microparticles (Cur--SLH) and blank silica-lipid hybrid microparticles (Blank-SLH). Results are shown as mean value±S.D. (*N*=3)

Viability of L929 murine fibroblast cells was measured by CCK-8 assay and the results are in Fig. 6. The preliminary cell studies demonstrated that the blank carriers do not have negative effect on the cell viability, which indicated high safety of the excipients used in the formula. Curcumin nanocarriers deterred the cell viability in a dose-dependent manner. The cell viability was over 80% when the concentration of nanocarriers was less than 45 μg/mL. The viability dropped to less than 50% when L929 murine fibroblast cells were treated with 150 μg/mL nanocarriers. The results indicate that curcumin at a certain concentration had evident cytotoxicity. In addition, the same concentration of curcumin microparticles was less cytotoxic than nanoemulsion when the concentration was higher than 30 μg/mL, which was probably due to the incomplete release of curcumin in the solid preparation.

## CONCLUSIONs

In this study, curcumin-loaded oil in water nanoemulsion and curcumin silica-lipid hybrid microparticles were successfully optimized to improve the solubility and bioavailability of curcumin. Orthogonal experiment was used to obtain an optimum emulsion composition. The microparticles were prepared using a two-step process, which showed good flow properties. XRD analysis suggested that curcumin was in XRD-amorphous instead of crystalline state in the microparticles. FTIR experiment showed that curcumin was encapsulated in Aerosil 380 and remained dissolved in the microparticles. *In vitro* antioxidant activity experiment confirmed that both formulations had good capacity for DPPH free radical scavenging and anti-lipid peroxidation. Results of *in vitro* digestion showed that the product digestion occurred mainly in intestinal juice due to the effects of bile salts and pancreatic enzymes. Compared with nanoemulsion, the rate and extent of free fatty acid release were reduced when the lipid was incorporated with Aerosil 380, which was due to the restraint of microparticles on the lipolysis of oil droplets. Moreover, the bioaccessibility of curcumin from nanoemulsion and microparticles was significantly higher than from curcumin carboxymethylcellulose sodium suspensions, which indicated that curcumin-lipid complexes could enhance the absorption of curcumin. The two formulations could maintain good chemical stability for 6 weeks at different temperatures in the dark and the microparticles protected curcumin better from degradation under light conditions. The curcumin carriers showed a low cell cytotoxicity when the concentration of curcumin was less than 45 μg/mL. It can be concluded that the solid preparation has better stability and is a promising approach to improve the bioavailability for curcumin.
